# Scrambled eggs: A highly sensitive molecular diagnostic workflow for *Fasciola* species specific detection from faecal samples

**DOI:** 10.1371/journal.pntd.0005931

**Published:** 2017-09-15

**Authors:** Nichola Eliza Davies Calvani, Peter Andrew Windsor, Russell David Bush, Jan Šlapeta

**Affiliations:** 1 Laboratory of Veterinary Parasitology, Sydney School of Veterinary Science, Faculty of Science, The University of Sydney, Camperdown, New South Wales, Australia; 2 Mekong Livestock Research Group, Sydney School of Veterinary Science, Faculty of Science, The University of Sydney, Camperdown, New South Wales, Australia; Queen's University Belfast, UNITED KINGDOM

## Abstract

**Background:**

Fasciolosis, due to *Fasciola hepatica* and *Fasciola gigantica*, is a re-emerging zoonotic parasitic disease of worldwide importance. Human and animal infections are commonly diagnosed by the traditional sedimentation and faecal egg-counting technique. However, this technique is time-consuming and prone to sensitivity errors when a large number of samples must be processed or if the operator lacks sufficient experience. Additionally, diagnosis can only be made once the 12-week pre-patent period has passed. Recently, a commercially available coprological antigen ELISA has enabled detection of *F*. *hepatica* prior to the completion of the pre-patent period, providing earlier diagnosis and increased throughput, although species differentiation is not possible in areas of parasite sympatry. Real-time PCR offers the combined benefits of highly sensitive species differentiation for medium to large sample sizes. However, no molecular diagnostic workflow currently exists for the identification of *Fasciola* spp. in faecal samples.

**Methodology/Principal findings:**

A new molecular diagnostic workflow for the highly-sensitive detection and quantification of *Fasciola* spp. in faecal samples was developed. The technique involves sedimenting and pelleting the samples prior to DNA isolation in order to concentrate the eggs, followed by disruption by bead-beating in a benchtop homogeniser to ensure access to DNA. Although both the new molecular workflow and the traditional sedimentation technique were sensitive and specific, the new molecular workflow enabled faster sample throughput in medium to large epidemiological studies, and provided the additional benefit of speciation. Further, good correlation (R^2^ = 0.74–0.76) was observed between the real-time PCR values and the faecal egg count (FEC) using the new molecular workflow for all herds and sampling periods. Finally, no effect of storage in 70% ethanol was detected on sedimentation and DNA isolation outcomes; enabling transport of samples from endemic to non-endemic countries without the requirement of a complete cold chain. The commercially-available ELISA displayed poorer sensitivity, even after adjustment of the positive threshold (65–88%), compared to the sensitivity (91–100%) of the new molecular diagnostic workflow.

**Conclusions/Significance:**

Species-specific assays for sensitive detection of *Fasciola* spp. enable ante-mortem diagnosis in both human and animal settings. This includes Southeast Asia where there are potentially many undocumented human cases and where post-mortem examination of production animals can be difficult. The new molecular workflow provides a sensitive and quantitative diagnostic approach for the rapid testing of medium to large sample sizes, potentially superseding the traditional sedimentation and FEC technique and enabling surveillance programs in locations where animal and human health funding is limited.

## Author summary

Fasciolosis caused by infection with *F*. *hepatica*, *F*. *gigantica* and their hybrid strains is an important health issue in medical and veterinary sciences. The economic impacts of infection and disease on production animals is of particular concern in low-income rural areas of developing countries, including in Southeast Asia where aquatic rice production plus low-input large ruminant husbandry, provides an ideal habitat for parasite proliferation. Here, we describe the development of a robust approach to ante-mortem diagnosis capable of differentiating between the two *Fasciola* spp. and their hybrid. Previously, such differentiation was based on identification of flukes collected post-mortem, because eggs of the two species have overlapping morphology. The major novelty is the demonstration that *Fasciola* spp. eggs, released by adults in the bile ducts, can be effectively and reproducibly broken and the content made available for DNA isolation. Coupled with part of a traditional sedimentation technique, this approach presents a new molecular diagnostic workflow capable of the specific detection of *F*. *hepatica* DNA in samples with ≤10 eggs per gram of faeces using a real-time PCR TaqMan assay. The presented workflow enables differentiation of *F*. *hepatica* and *F*. *gigantica* and their hybrid if a duplex TaqMan real-time PCR assay is included. The ability to process medium to large sample sizes *in lieu* of a continuous cold chain will enable further research into the epidemiology, control and public health concerns associated with *Fasciola* spp. infection.

## Introduction

Fasciolosis, due to *Fasciola hepatica* and *Fasciola gigantica*, is an important zoonotic production-limiting disease of ruminants [1]. In 2005, an estimated 91 million people across 8 countries were considered at risk of contracting this neglected tropical disease, with children the most likely to become infected [2]. Despite the number of people at risk, fasciolosis is generally considered a neglected disease of humans due to its chronic nature and subsequent underreporting [3]. Livestock and wildlife act as definitive hosts, although it has been demonstrated that humans may also play a participatory role in the spread of the parasite [4]. Human infection typically occurs through the ingestion of freshwater plants such as watercress, although infection via consumption of metacercariae-contaminated water has also been documented [5].

Infections are traditionally diagnosed via the sedimentation of faecal samples, or more recently through immunological tests such as a commercially available coprological antigen ELISA (coproELISA) [6, 7]. Neither of these methods can differentiate *F*. *hepatica* from *F*. *gigantica* or *vice versa* [8]. A high degree of operator error is associated with sedimentation outcomes, with faecal egg count (FEC) results often differing between operators, depending on their level of experience. Species identification by extraction of adult flukes requires post-mortem analysis of the liver, which is not possible in human cases and often of limited availability in ruminants unless conducted during abattoir surveillance. Other non-invasive forms of diagnosis in humans and animals, including serological techniques, are unable to provide species confirmation [7]. DNA isolation and real-time PCR analysis have the potential for a preferred diagnostic solution, offering increased throughput, reproducibility, and higher sensitivity, with the added benefit of species differentiation. Despite this potential, no published method currently exists for the reliable, highly sensitive and specific diagnosis of infection from faecal samples.

The aim of this study was to develop a new molecular diagnostic workflow for real-time PCR detection of *F*. *hepatica* eggs in ruminant faecal samples and to subsequently compare the results to a traditional sedimentation diagnostic test. The workflow involves an optimised disruption protocol to isolate DNA from *Fasciola* spp. eggs, and a real-time PCR assay that was evaluated for sensitivity and specificity. Additionally, DNA isolation and real-time PCR of a partial and whole pellet from a traditional sedimentation was tested to determine if these additional steps improved the analytical sensitivity of the PCR assay. Finally, the diagnostic sensitivity and specificity of the coproELISA was evaluated in comparison to the traditional sedimentation and newly-developed molecular diagnostic workflow. The positive cut-off threshold was assessed in order to increase the sensitivity and specificity of the ELISA for samples with low egg counts (≤10 eggs per gram, EPG). The end result is a new molecular workflow for the diagnosis of *F*. *hepatica* that was applied to samples from a cohort of beef cattle with constant *F*. *hepatica* exposure. Further, the impact of sample storage in 70% ethanol (EtOH) on sedimentation outcomes and DNA isolation and amplification were assessed to determine the feasibility of transporting samples in situations where cold chains may not be readily available. The development of a new molecular diagnostic workflow would enable the highly sensitive detection of *Fasciola* spp. for the quantification of faecal egg load and species identification in regions of parasite sympatry, such as Southeast Asia.

## Materials and methods

### Faecal samples

#### *Fasciola hepatica* infected sheep faecal samples

Faecal samples from Merino sheep (n = 10) positive for *F*. *hepatica* were collected from a property near Kemps Creek in New South Wales in October 2016 during routine animal handling by a licensed veterinarian. Samples were submitted for parasitological diagnostics (Sydney School of Veterinary Science, The University of Sydney) where they were stored at 4°C for up to two weeks prior to processing.

#### *Fasciola hepatica* infected cattle faecal samples

Faecal samples (n = 31) from Murray Grey beef cattle greater than two years of age suspected to have been exposed to *F*. *hepatica* infection during a flooding event in 2015 were collected five months apart (October 2016 and February 2017; spring and summer, respectively (S1 Table)). The grazing cattle were located on a property near Newcastle (Herd 1), New South Wales, and submitted for parasitological diagnostics (Sydney School of Veterinary Science, The University of Sydney). No flukicide treatment had previously occurred on the property. A further 10 samples were submitted for parasitological examination in March 2017 from a cohort of mixed beef cattle (Brangus, Charolais) on a nearby property in Newcastle (Herd 2), New South Wales, with endemic fasciolosis. Herd 2 had been treated with triclabendazole six months prior to sample collection. All samples were collected by a licensed veterinarian during routine animal handling for diagnostic purposes (Sydney School of Veterinary Science, The University of Sydney). All animals from both Herd 1 and 2 had concurrent infections with paramphistome species (Bovine stomach fluke). Samples were stored at 4°C for up to four weeks prior to processing.

#### *Fasciola gigantica* infected cattle faecal samples

Faecal samples (n = 5) positive for *F*. *gigantica* from local native cattle in Cambodia were collected in February 2017 during routine animal health checks by a licensed veterinarian and stored in 70% EtOH before being shipped to Australia for real-time PCR analysis (Sydney School of Veterinary Science, The University of Sydney).

### Sedimentation and faecal egg count

FECs were determined by a standard faecal sedimentation method with minor modifications as follows [9]. Faecal samples (3 g and 6 g for sheep and cattle, respectively) were mixed with distilled water to form a homogenous solution. The solution was hosed with tap water through a 270 μm nylon sieve into a 250 ml conical measuring cylinder, topped with distilled water and allowed to sediment for three minutes. After three minutes the supernatant was aspirated and the sediment poured into a 100 ml conical measuring cylinder, topped with distilled water and allowed to sediment for a further three minutes. Again, the supernatant was aspirated and the remaining sediment poured into a 15 ml centrifuge tube, where it was once more topped with distilled water and allowed to sediment for a final three minutes. The supernatant was aspirated and discarded, leaving 2 ml of sediment which was thoroughly vortexed to ensure homogeneity. To examine presence of fluke eggs, 2 drops of methylene blue (1%) was added to the sediment and examined under an Olympus LG-PS2 stereomicroscope using a 6.5×17×1 cm grid tray at 15× magnification. An additional 20 ml of distilled water was added to the tray to allow ease of counting. Each faecal sample was sedimented and counted in duplicate, resulting in a total of 6 g and 12 g being counted for individual sheep and cattle, respectively. All yellow-brown *Fasciola* spp. eggs were counted.

Triplicate clean faecal samples were spiked with a known number of *F*. *hepatica* eggs and the percentage lost during the sedimentation process was calculated. The results were in agreement with the original protocol and demonstrated that one third of eggs from the initial sample volume were retained in the sediment after processing [9]. Hence the final number of eggs from individual cattle sedimentations was divided by 2 to obtain EPG (S2 Table). All EPGs are presented as a mean of the two independent sedimentations. All sedimentations were conducted by the same technician to remove any variability in counting. The technician was unaware of the previous results to prevent bias between replicate counts.

### Adult *Fasciola* spp. samples

Clean adult *F*. *hepatica* and *F*. *gigantica* flukes stored in 70% EtOH from the parasite collection at the Sydney School of Veterinary Science, University of Sydney were used as positive controls. Total genomic DNA from 1/5^th^ of an adult fluke (25 mg, cut using a sterile scalpel blade) from each species was isolated using Isolate II Genomic DNA kit (BioLine, Australia) according to the manufacturer’s instructions and eluted in 100 μl of elution buffer (10 mM TrisCl buffer, pH = 8.5). To monitor DNA isolation efficiency and PCR inhibitors 5 μl of DNA Extraction Control 670 (Bioline, Australia) was included and DNA assayed for presence of extraction control signal on CFX96 Touch Real-Time PCR Detection System with the corresponding CFX Manager 3.1 software (BioRad, Australia) using SensiFAST Probe No-ROX Mix (BioLine, Australia) according to the manufacturer's instructions with expected C_T_ values of <31. Each DNA isolation batch included a blank sample (ddH_2_O) to detect any potential contamination during the extraction process (extraction negative control). Extracted DNA was stored at -20°C prior to molecular analysis.

### Real-time PCR efficiency

A set of genus-specific primers were used to specifically amplify *Fasciola* spp. ITS2 rDNA region [10]. The real-time PCR utilised primers SSCP*Fa*F [S0754] (5′-TTG GTA CTC AGT TGT CAG TGT G-3′) and SSCP*Fa*R [S0755] (5′-AGC ATC AGA CAC ATG ACC AAG-3′) generating 140 bp amplicons [10]. *F*. *hepatica* species specific TaqMan probe ProFh [S0770] (5’-ACC AGG CAC GTT CCG TCA CTG TCA CTT T-3’) and *F*. *gigantica* specific TaqMan probe ProFg [S0771] (5’-ACC AGG CAC GTT CCG TTA CTG TTA CTT TGT-3’) were then implemented [10]. Use of probes removed non-specific background amplification detected using SYBR chemistry and provided species-specific confirmation (S1 Fig). The real-time PCR does not amplify paramphistome egg DNA isolated from cattle faecal samples (Herd 2) with concurrent infections (S2 Fig). The TaqMan probes were labelled with a 5’-FAM, 5’-HEX reporter dye, respectively, and 3’-BHQ1 quencher. The assay with Australian samples was run only with FAM labelled ProFh probe because *F*. *gigantica* is exotic to Australia. All primers and probes were from Macrogen Ltd. (Seoul, Korea). The real-time PCR reactions used SensiFAST Probe No-ROX Mix (BioLine, Australia) on CFX96 Touch Real-Time PCR Detection System with the corresponding CFX Manager 3.1 software (BioRad, Australia). The volumes of the real-time PCR reactions were made up to 20 μl, including 2 μl of template DNA. The PCR mix included primers and probes at final concentrations of 400 nM and 100 nM, respectively. PCR reactions were initiated at 95°C for 3 min, followed by 40 cycles of 5 s at 95°C and 10 s at 60°C. All runs were performed in duplicate and ddH_2_O acted as a negative control. The efficiency, limit of detection and limit of quantification of the real-time PCR was determined via seven serial 10-fold dilutions of the positive control (adult *F*. *hepatica*, 174.6 ng/μl measured with a NanoDrop Nd-1000 spectrophotometer, Thermo Scientific, Australia), representing a range in concentration of 1.75 x 10^1^ to 1.75 x 10^−5^ ng/μl. Results were considered to be positive if both replicates displayed C_T_ values <36.

### Optimisation of sample disruption conditions

#### Disruption of clean eggs

Clean *F*. *hepatica* eggs in phosphate buffered saline (pH = 7.4) (PBS) were covered to prevent exposure to light and stored at 4°C for up to two months prior to processing. Total genetic DNA was isolated from the clean eggs using Isolate Faecal DNA kit (BioLine, Australia), with the following modification to the initial homogenisation step. A total of 2000 eggs in 150 μl of PBS were added to the 2 ml DNA isolation kit homogenisation tube with 750 μl of lysis buffer (Isolate Faecal DNA kit, BioLine) and 5 μl of DNA Extraction Control 670 (Bioline, Australia) and homogenised using a high-speed benchtop homogeniser FastPrep-24 (MP Biomedicals, Australia) under the following conditions; (i) 1 × 40 seconds at 4.0 m/s, (ii) 2 × 40 seconds at 4.0 m/s, (iii) 3 × 40 seconds at 4.0 m/s, (iv) 1 × 40 seconds at 6.0 m/s, (v) 2 × 40 seconds at 6.0 m/s and (vi) 3 × 40 seconds at 6.0 m/s, with 5 minute breaks between each 40 second run, at which time the samples were stored on ice. Samples were disrupted and isolated in duplicate for each of the six treatments (i.–vi.) An aliquot of each sample was examined under a light microscope (Olympus BX41, Australia) at 200 × magnification to ensure no intact eggs remained, before being isolated for DNA amplification.

#### Disruption of eggs in faecal samples

Total genetic DNA was isolated from 150 mg sheep faecal samples with known concentrations of *F*. *hepatica* eggs (267 EPG) using Isolate Faecal DNA kit (BioLine, Australia) into 100 μl elution buffer (10 mM TrisCl buffer, pH = 8.5), with the following modification to the initial homogenisation step. Samples were added to the 2 ml DNA isolation kit homogenisation tube with 750 μl of lysis buffer (Isolate Faecal DNA kit, BioLine) and 5 μl of DNA Extraction Control 670 (Bioline, Australia) and homogenised using a high-speed benchtop homogeniser FastPrep-24 (MP Biomedicals, Australia) under the following conditions; (iv) 1 × 40 seconds at 6.0 m/s, (v) 2 × 40 seconds at 6.0 m/s and (vi) 3 × 40 seconds at 6.0 m/s with 5 minute breaks between each 40 second run, at which time the samples were stored on ice. All subsequent isolations from cattle samples of unknown *F*. *hepatica* status (Herd 1 and 2) were disrupted at 6.0 m/s for 40 seconds (disruption condition iv.) on the FastPrep-24 (MP Biomedicals, Australia), after which time they were stored on ice until isolation.

For the isolation of DNA from single *F*. *hepatica* and *F*. *gigantica* eggs, cattle faecal samples, (Newcastle, Australia and Takeo, Cambodia, respectively), were sedimented and individual eggs were manually removed (with a 20 μl pipette) and placed into 2 ml DNA isolation kit homogenisation tubes with 750 μl lysis buffer (Isolate Faecal DNA kit, BioLine) and 5 μl of DNA Extraction Control 670 (Bioline, Australia). DNA was isolated from five replicates of individual eggs from each species using disruption condition iv. (6.0 m/s for 40 seconds).

### Molecular genotyping of *F*. *hepatica*, *F*. *gigantica* and paramphistomes

Three conventional PCR assays were used to confirm adult *Fasciola* spp. (Adult *Fasciola* spp. samples) [11, 12]. The internal transcribed spacers 1 (ITS1) and 2 (ITS2) were amplified using primers ITS1-F [S0762] (TTG CGC TGA TTA CGT CCC TG) and ITS1-R [S0763] (TTG GCT GCG CTC TTC ATC GAC) and ITS2-F [S0764] (TGT GTC GAT GAA GAG CGC AG) and ITS2-R [S0765] (TGG TTA GTT TCT TTT CCT CCG C), yielding 639-bp and 519-520-bp-long fragments, respectively [11]. DNA fragments of a 577-bp-long 28S rDNA sequence were amplified using primers 28F1 [S0756] (ACG TGA TTA CCC GCT GAA CT) and 28R600 [S0757] (CTG AGA AAG TGC ACT GAC AAG) [12]. The primers targeting *Fasciola* spp. ITS2 were also used to amplify DNA from paramphistome eggs collected from cattle faecal samples with concurrent infections (Herd 2) [11].

All PCR amplifications were performed with MyTaq Red Mix (BioLine, Australia) in a total volume of 30 μl. Primers were added at a concentration of 250 nM each. The PCR was run using the following cycling conditions: 95°C for 15 s, 55°C for 15 s and 72°C for 20 s for 35 cycles. All reactions were initiated at 95°C for 2 min and concluded at 72°C for 7 min. PCRs were amplified in the Verity PCR cycler (Thermo Fisher Scientific, Australia). Each PCR reaction contained 2 μl of sample DNA. All PCRs were run with negative controls (ddH2O). All PCRs that yielded unambiguous single bands of the expected size were directly and bidirectionally sequenced using amplification primers at Macrogen Ltd. (Seoul, Korea) and assembled and compared to reference sequences for *F*. *hepatica* and *F*. *gigantica* (AB207139 and AB207143, respectively) in CLC Main Workbench 6.9.1 (Qiagen, CLC Bio) [11].

### Diagnostic application

**Fig 1:**
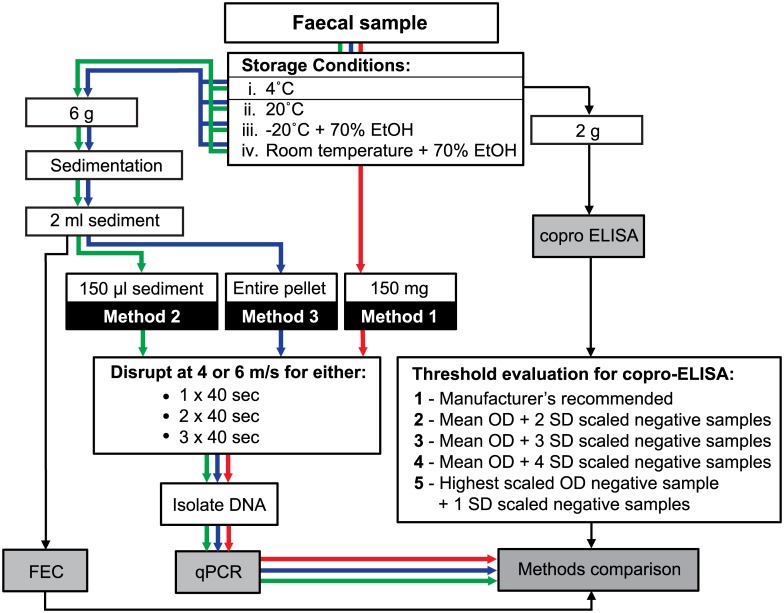
Comparison of three diagnostic methods for the diagnosis of fasciolosis in cattle faecal samples. A traditional sedimentation and faecal egg count (FEC) was performed and the results compared to a coproELISA and real-time PCR. Three methods of sample preparation were compared; isolation of DNA from 150 mg raw faeces (Method 1; red), sedimentation followed by isolation of DNA from 150 μl of the resultant sediment (Method 2; green), and sedimentation followed by isolation of DNA from the entire sediment pellet (Method 3; blue). Egg disruption prior to DNA isolation was assessed using six methods on a benchtop homogeniser using either 4.0 or 6.0 m/s for 1, 2 or 3 rounds of beating for 40 seconds between which samples were stored on ice. The manufacturer-recommended positive threshold of a commercially-available coproELISA was compared to four others suggested in the literature; mean scaled optical density (OD) of negative samples + 2 × standard deviations (SD) of scaled negative samples (Threshold 2) [13], mean scaled OD of negative samples + 3 × SD of scaled negative samples (Threshold 3) [13–15], mean scaled OD of negative samples + 4 × SD of scaled negative samples (Threshold 4) [13], and highest scaled OD of negative samples + 1 SD of negative samples (Threshold 5) [16].

Three sample preparation methods (Fig 1; workflows red [Method 1], green [Method 2] and blue [Method 3]) were used to compare the diagnostic sensitivity and specificity of the real-time PCR on a naturally infected herd with low FECs (Herd 1). For each animal, DNA from 150 mg (Method 1) pure faeces was isolated using the Isolate Faecal DNA kit (BioLine, Australia) and disruption condition iv. Method 2 consisted of a modification to the sedimentation procedure where the final 2 ml of sediment was vortexed to ensure thorough mixing before 150 μl (≈150 mg) was removed and DNA isolated using disruption condition iv. A further modification of the sedimentation procedure was employed in Method 3 where the entire 2 ml (≈2 g) of sediment was centrifuged at 2500 g for 10 minutes to form a pellet of concentrated eggs. The entire pellet was manually removed from the 15 ml centrifuge tube using a combination of Pasteur pipettes and fine wooden applicator sticks for DNA isolation using disruption condition iv. The diagnostic sensitivity and specificity of Method 3 was further confirmed on a herd of cattle with constant *F*. *hepatica* exposure (Herd 2).

The analytical sensitivity of the three sample preparation methods was determined by comparing the morphological FEC from the duplicate traditional sedimentations (henceforth referred to as mEPG) to the FEC calculated according to the standard curve produced by the optimised real-time PCR (henceforth referred to as qEPG).

### Impact of storage conditions on sedimentation outcomes and DNA isolation efficiency

To determine the effect of storage conditions on sedimentation and DNA isolation outcomes, 6 g aliquots of clean cattle faecal samples spiked with 2000 *F*. *hepatica* eggs were stored under the following conditions for one month; i. 4°C, ii. -20°C, iii. -20°C + 70% EtOH, iv. room temperature + 70% EtOH. Samples were spiked and stored in duplicate. After a month, the EtOH was aspirated and samples were placed in an incubator at 37°C to dry. After drying, each sample was sedimented and counted as described in previously and DNA was isolated as described in the section on diagnostic application (Method 3) under disruption condition iv.

### Comparison with a commercially-available coproELISA for detection of *F*. *hepatica* antigen in faeces

Presence of *F*. *hepatica* antigen in faecal samples was assayed using ‘Monoscreen AgELISA *Fasciola hepatica*’ (BIO K 201, Bio-X Diagnostics S.A., Belgium) (coproELISA), an indirect sandwich ELISA kit for the detection of *Fasciola* spp. antigen in cattle and sheep faeces (Fig 1). Cattle faecal samples (Herd 1 and 2) were mixed with the kit dilution buffer 1:1 (2 g + 2 ml) in 12 ml centrifuge tubes and vortexed for 30 seconds until thoroughly mixed followed, by centrifugation for 10 minutes at 2500 g. The supernatant (0.5 ml) was aspirated and stored in labelled microcentrifuge tubes at 4°C until analysis. There was no effect on positive/negative coproELISA outcomes for samples processed immediately or stored at 4°C for up to 96 h (S3 Fig; [15]). The coproELISA was performed according to manufacturer instructions with 100 μl of supernatant (prepared as above). Each batch included two positive reference samples as controls. Optical densities (OD) were read at 450 nm using a SpectraMax 250 plate reader (Molecular Devices, LLC., Sunnyvale CA, USA). ODs of each corresponding negative well was subtracted from the individual sample ODs (Net OD). The Scaled OD was calculated by dividing the Net OD of the sample by the Net OD of the positive coproELISA controls. Samples were considered positive for *F*. *hepatica* antigen if the scaled OD was >0.08 (Monoscreen AgELISA *Fasciola hepatica*, BIO K 201 batch number FASA16B23).

### Statistical analysis

As a post-mortem analysis was unable to be performed to confirm the presence or absence of flukes in the liver, the sedimentation technique was considered the gold standard and the diagnostic sensitivity and specificity of the coproELISA and real-time PCR methods were calculated accordingly. Data was analysed in Microsoft Excel (2013) and visualised with GraphPad Prism version 6 (GraphPad Software, USA).

### Data accessibility

Nucleotide sequences have been deposited in GenBank (ITS1: MF678648—MF678649; ITS2: MF678650—MF678652; 28S: MF678653—MF678654). The final protocol has been deposited online on protocols.io and can be found at https://dx.doi.org/10.17504/protocols.io.jggcjtw. Raw and supplementary data related to this article have been deposited online on Mendeley Data and can be found under the following DOI’s; https://dx.doi.org/10.17632/9zfzv84p8f.2 and https://dx.doi.org/10.17632/4gwjjk47sz.3, respectively.

## Results

### Short periods of bead-beating provide consistent *F*. *hepatica* egg rupture for efficient DNA isolation and amplification

Visual inspection determined that all *F*. *hepatica* eggs were disrupted after a single round of homogenisation for 40 seconds at 6.0 m/s for both clean eggs and sheep faecal samples. Real-time PCR yielded similar C_T_ values (15.5–16.6) for all six egg disruption conditions when applied to 2000 clean *F*. *hepatica* eggs in 150 μl PBS (Table 1). Similarly, when applied to 150 mg of *F*. *hepatica* infected sheep faecal samples (267 EPG, equivalent to 40 eggs in 150 mg), similar C_T_ values (21.2–22.0) were observed (Table 2).

**Table 1:** C_T_ values of 2000 clean *F*. *hepatica* eggs in phosphate buffered saline (PBS) when subjected to six disruption treatments.

	Treatment						
Replicate	i.	ii.	iii.	iv.	v.	vi.	SD
1	16.61	16.23	15.96	16.07	16.14	15.69	0.28
2	16.26	16.04	15.49	16.18	15.65	15.77	0.28

i. 1 × 40 seconds at 4.0 m/s; ii. 2 × 40 seconds at 4.0 m/s; iii. 3 × 40 seconds at 4.0 m/s; iv.1 × 40 seconds at 6.0 m/s; v. 2 × 40 seconds at 6.0 m/s; vi. 3 × 40 seconds at 6.0 m/s.

**Table 2:** C_T_ values of *F*. *hepatica* eggs in sheep faecal samples when subjected to three different disruption treatments.

	Treatment			
Replicate	iv.	v.	vi.	SD
1	21.58	21.17	21.74	0.24
2	21.48	21.21	21.96	0.31

iv.1 × 40 seconds at 6.0 m/s; v. 2 × 40 seconds at 6.0 m/s; vi. 3 × 40 seconds at 6.0 m/s.

The real-time PCR assay was highly efficient (100%, R^2^ = 0.995) at detecting *F*. *hepatica* DNA from adult fluke samples. The initial value of the 10-fold serial dilution of *F*. *hepatica* DNA (1.75 x 10^1^ ng/μl measured with a NanoDrop ND-1000 spectrophotometer, Thermo Scientific, Australia) gave a corresponding C_T_ value to the 2000 clean eggs in 150 μl PBS (S4 Fig).

The standard curve derived from the serial 10-fold dilution gave intervals of 3.4 C_T_ values for concentrations of 1.75 x 10^1^ to 1.75 x 10^−5^ ng/μl (S5 Fig). Henceforth, the *F*. *hepatica* adult fluke DNA dilution was considered a positive reference and was used to determine qEPG values. The assay routinely detected concentrations of 1.75 x 10^−4^ mg pure *F*. *hepatica* DNA (equivalent to a theoretical limit of 2 x 10^−2^ eggs), demonstrating the limit of quantification and occasionally detected 1.75 x 10^−5^ mg (equivalent to a theoretical limit of 2 x 10^−3^ eggs), giving the limit of detection. This theoretical limit was tested in practice through the isolation of DNA from single *F*. *hepatica* and *F*. *gigantica* eggs. DNA from five individual eggs from each species was isolated and amplified in duplicate. For both species, 9/10 wells amplified, giving the analytical sensitivity and further demonstrating the value of the bead-beating technique (Table 3).

**Table 3:** C_T_ values from DNA isolation of single *F*. *hepatica* and *F*. *gigantica* eggs subjected to 40 seconds of homogenisation at 6.0 m/s (disruption condition iv.).

Sample ID	1		2		3		4		5		
Replicate	1	2	1	2	1	2	1	2	1	2	Mean Ct (SD)
***F*. *hepatica***	35.16	34.23	34.35	37.67	37.83	-	32.18	32.68	35.29	34.99	34.93 (1.81)
***F*. *gigantica***	35.40	35.36	35.16	35.10	36.02	35.72	38.15	-	34.42	34.72	35.51 (1.07)

### A new molecular workflow for the sensitive detection of *Fasciola* spp. eggs in faeces

**Fig 2:**
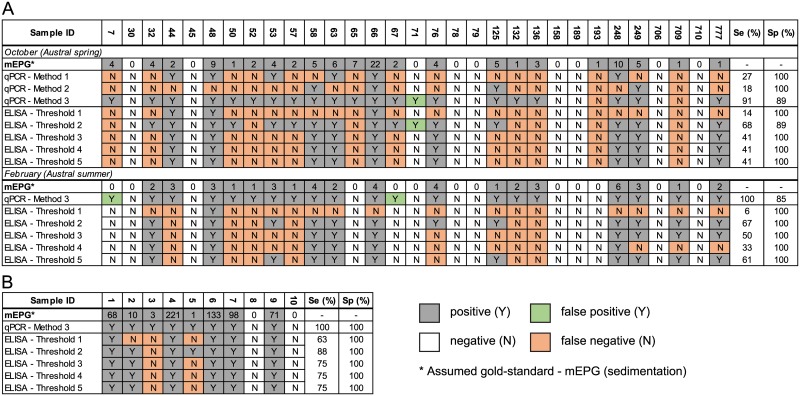
Comparison of the sensitivity (Se) and specificity (Sp) of three methods for the diagnosis of fasciolosis in cattle faecal samples. (A) FEC results presented as the mean mEPG of two sedimentations in comparison to three methods for real-time PCR analysis and five positive threshold cut-offs for a commercially-available coproELISA for a herd of cattle exposed to fluke two years prior, sampled during the Austral spring and summer. (B) As for A with only one real-time PCR method (Method 3) used on a herd of cattle with endemic fasciolosis. Positive and negative results for each method and threshold are indicated by a Y or N, respectively. Using the FEC data as the assumed gold standard, false negative results are indicated by orange squares, and false positive results are indicated by green squares. Individual animal IDs are presented in the top row for both A and B.

**Fig 3:**
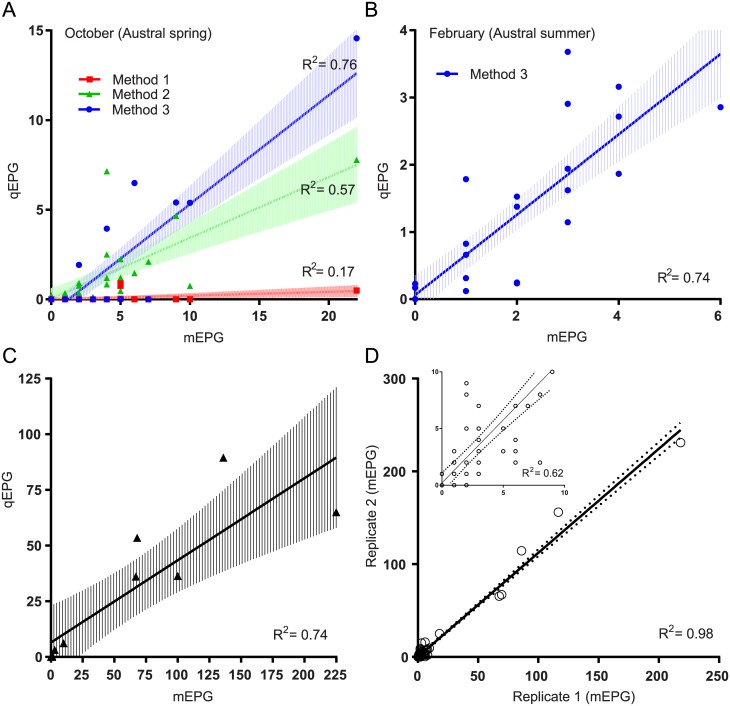
Correlation between faecal egg counts and real-time PCR egg estimates. (A) Real-time PCR egg estimates (qEPG) for three methods of sample preparation for DNA isolation from cattle samples collected during Austral spring (Herd 1) are compared to morphological FEC (mEPG); isolation of DNA from 150 mg raw faeces (Method 1), sedimentation followed by isolation of DNA from 150 μl of the resultant sediment (Method 2), and sedimentation followed by isolation of DNA from the entire sediment pellet (Method 3). (B) As for A using only Method 3 on samples collected during Austral summer (Herd 1). (C) As for B using samples collected from an endemic population (Herd 2). (D) Correlation between duplicate sedimentations (mEPG) for all samples (Herds 1 and 2) across all sampling periods; inset–correlation between duplicate FECs for samples with ≤ 20 EPG.

The three methods of sample preparation were compared for detection of *F*. *hepatica* DNA in faecal samples with low FECs (≤10 EPG). Method 3 proved the most sensitive after disruption at 6.0 m/s for 40 seconds (disruption condition iv.), demonstrating 91–100% diagnostic sensitivity in comparison to the traditional sedimentation technique and FEC in cattle (n = 31) with low FECs (Herd 1) (Fig 2A). Method 3 involved isolating DNA from the entire pellet from a traditional sedimentation, which occasionally exceeded the maximum manufacturer-recommended volume of 150 mg. However, no impact of increased sample volume on DNA isolation and amplification was detected (S3 Table). Method 3 was then used to diagnose *F*. *hepatica* infection in a cattle herd (n = 10) with constant *F*. *hepatica* exposure (Herd 2) where 100% diagnostic sensitivity was observed again (Fig 2B). Additionally, Method 3 showed good correlation (0.74–0.76) with FECs for all herds and sampling periods (Fig 3A–3C). In comparison, Methods 1 and 2 show poorer correlation (0.17 and 0.57, respectively) (Fig 3A).

### Comparison of the diagnostic sensitivity and specificity of sedimentation (mEPG), real-time PCR (qEPG) and coproELISA diagnostic tests on field samples

More than half (71% and 58%, October 2016 and February 2017, respectively) of Herd 1 (n = 31) was positive for *F*. *hepatica* by sedimentation on both collection dates (Fig 2A and Table 4). The average FEC result for Herd 1 during the Austral spring and summer was 5 EPG (±4.55) and 3 EPG (±1.34), respectively (Table 4). For Herd 2, 80% (8/10) of the animals were positive for *F*. *hepatica* by faecal sedimentation and the average FEC was 61 EPG (±70.25) (Fig 2B and Table 4). A strong positive correlation (0.98) was observed between duplicate FECs (Fig 3D).

**Table 4:** *F*. *hepatica* faecal egg counts (FEC) from two cattle herds located in Newcastle, New South Wales.

	Herd 1		Herd 2
	October 2016	February 2017	March 2017
**Total Samples**	31	31	10
**Total Positive (%)**	22 (71)	18 (58)	8 (80)
**Mean EPG (±SD)**	4.59 (±4.55)	2.56 (±1.34)	60.50 (±70.25)

A commercially available coproELISA was used to diagnose *F*. *hepatica* infection in both Herd 1 and 2 across all sampling periods and the diagnostic sensitivity and specificity at five different thresholds was calculated (Fig 2). Threshold 2 proved to be the most sensitive cut-off and was calculated by averaging the scaled OD and adding two standard deviations of the known negative samples [13]. Using this cut-off the diagnostic sensitivity ranged from 65–88% for both herds over all sampling periods. In comparison, the manufacturer’s recommended cut-off yielded a larger range in diagnostic sensitivity of 6–63% for both herds over the two sampling periods (Fig 2).

When using the new molecular workflow (disruption condition iv. and sample preparation Method 3) as the gold standard of *Fasciola* spp. diagnosis, the diagnostic sensitivity and specificity of the traditional sedimentation technique is 90–100% and 80–100%, respectively (S4C Table). In comparison, the diagnostic sensitivity and specificity of the coproELISA at Threshold 2 is 60–88% and 100%, respectively (S4D Table).

### Differentiation of *F*. *hepatica* from *F*. *gigantica* using DNA isolated from eggs in faeces

The DNA isolated using disruption protocol iv. was suitable for the differentiation between *F*. *hepatica* and *F*. *gigantica* for both conventional and real-time PCR. *F*. *hepatica* DNA isolated from cattle faecal samples (Herd 1) was used to successfully amplify and sequence a 28S rDNA gene fragment 100% matching our reference 28S rDNA (MF678654). The 28S rDNA gene fragment is 99.5% identical (3 nt differences across 577 nt) between *F*. *hepatica* (MF678654) and *F*. *gigantica* (MF678653). Primers targeting ITS1 and ITS2 are not *Fasciola* spp. specific i.e. they also amplify paramphistome DNA, therefore were not used in genotyping PCRs with DNA isolated from faecal samples.

### Storage condition has no effect on DNA isolation outcomes enabling transport of samples in the absence of a complete cold chain

Storage conditions had no effect on sedimentation, DNA isolation and amplification outcomes (Table 5). Regardless of the storage condition applied, sedimentation results remained consistent across all replicates and DNA amplification remained unaffected (S5 Table).

**Table 5:** C_T_ values of samples subjected to different storage conditions prior to sedimentation and DNA isolation.

	Treatment				
Replicate	4°C	-20°C	-20°C + 70% EtOH	Room Temperature + 70% EtOH	SD
1	22.42	22.13	21.35	22.32	0.42
2	22.85	21.77	21.67	21.85	0.26

## Discussion

A new molecular workflow was developed in response to the need for a specific diagnostic tool for *Fasciola* spp. in faecal samples [17–19]. The technique enabled medium to large sample throughput with high sensitivity in order to detect changes in faecal *Fasciola* spp. egg load, with the added benefit of parasite speciation (i.e. *F*. *hepatica* vs *F*. *gigantica*). In the present study, the extraction of DNA from *F*. *hepatica* eggs through the use of a bead-beating approach resulted in consistent DNA isolation. The bead-beating approach (iv.) was applied to raw faecal samples with low *F*. *hepatica* EPG, but demonstrated decreased diagnostic sensitivity. To improve the diagnostic sensitivity, a *Fasciola*-egg concentration technique through egg sedimentation (Method 3) was combined with the bead-beating approach prior to DNA isolation. The new molecular workflow was highly sensitive (91–100%) with the real-time PCR results showing good correlation with faecal egg counts (0.74–0.76), enabling robust quantitative detection of *Fasciola* species-specific eggs in faeces.

The hard shell of *Fasciola* spp. eggs must first be disrupted to ensure access to the inner contents for DNA isolation and subsequent amplification. Although mechanical disruption prior to DNA extraction has been previously employed for the isolation of *Fasciola* spp. DNA from faeces, demonstration of the efficiency of mechanical disruption is lacking [20–22]. Our study shows consistent mechanical rupture of *F*. *hepatica* eggs across a wide range of settings on a high-speed benchtop homogeniser. Our results of mechanical egg disruption are consistent with findings for other parasitic species with notoriously robust eggs, such as *Trichuris trichiura* and *Echinococcus multilocularis* [23–24]. The DNA isolation protocol successfully isolated DNA from single *F*. *hepatica* and *F*. *gigantica* eggs using a single short 40s period of bead-beating (disruption condition iv.). It has previously been shown that isolation of DNA from a single *Fasciola* spp. egg was possible after vortexing with glass beads for 30 minutes [21]. In a diagnostic laboratory setting, a rapid sample preparation and DNA isolation approach is paramount to take advantage of fast real-time PCR assays to deliver reproducible and accurate diagnostic results. The wide range of settings capable of disrupting *Fasciola*-eggs (disruption conditions i.–iv.) allowed for the selection of a bead-beating protocol that best aligns with other faecal sample molecular diagnostic procedures. As our laboratory utilises a standard protocol that includes 40s at speed of 6.0 m/s using FastPrep-24 (MP Biomedicals, Australia), this was incorporated into the new molecular workflow for *Fasciola* spp. DNA diagnostics. With the demonstration of a wide range of suitable settings able to disrupt *Fasciola*-eggs for DNA isolation, adaptation of our workflow for other laboratories will be appropriate.

Chronic *Fasciola* spp. infection in animals and humans give variable egg outputs, therefore we optimised our workflow to maximise the analytical sensitivity to <10 EPG [9,25–27]. Our initial success with clean eggs demonstrated consistent DNA isolation, although applying this approach to naturally-infected faecal samples with low EPGs (<10 EPG) proved challenging. When using the manufacturer-recommended maximum volume of raw faecal material (150 mg) the theoretical sensitivity is 6.67 EPG, assuming that each 150 mg of raw faecal material contains 1 *Fasciola* spp. egg (Method 1). This is in agreement with other work [28] reporting the analytical sensitivity of one *F*. *gigantica* egg in 100 mg faeces, equivalent to 10 EPG. Thus, Method 1 is of limited diagnostic use in chronically infected cattle that frequently report low egg numbers (≤10EPG) [25,27]. To improve the approach, a concentration step was included which employed the traditional sedimentation technique for trematode eggs [9] without the microscopic observation and counting (Method 3). A previously-described alternative method for egg concentration [21] used laborious washing and sieving procedures, including overnight refrigeration of a faecal suspension, making it inappropriate for application in diagnostics [29]. Our new molecular workflow incorporates a *Fasciola*-egg concentration procedure prior to the optimised *Fasciola*-egg disruption protocol, leading to highly successful results of 91–100% diagnostic sensitivity in a pilot study in cattle with ≤10 EPG.

To ensure that the diagnostic sensitivity of the optimised workflow was consistent across a range of faecal egg loads we tested our approach on ten samples from an endemically infected herd that had received a triclabendazole oral drench six months prior to sampling. Despite the smaller sample size, these samples were considered suitable for proof of principal of the optimised workflow due to their considerably larger EPG range (max. 221EPG, mean 61EPG) and our previous success across two time points in a herd with low EPGs. To our knowledge only two other studies [20,22] report results testing the capability of molecular diagnostic tools for the identification of *Fasciola* spp. infection in individual naturally infected animals. In contrast, the diagnostic capacity of our optimised workflow remained high [20]. The clear benefits of the concentration of eggs in samples prior to isolation address the diagnostic sensitivity limitations previously highlighted [20]. No details were provided in other work [22] regarding the sensitivity of the diagnostic approach on naturally infected samples. However, we maintain that our consistent results across different groups of naturally infected animals demonstrate the robustness of our approach to sample preparation for DNA isolation, regardless of the molecular tools employed. Hence the clear benefits of the new molecular workflow addresses the needs of the animal and human health industry in regards to increasing the analytical and diagnostic sensitivity of *Fasciola* spp. molecular diagnosis.

New antigen detection techniques for the diagnosis of *Fasciola* spp. infection (coproELISA) have been used to address the limitations of the traditional sedimentation and FEC approach by detecting infection prior to the completion of the pre-patent period [30]. We compared the diagnostic sensitivity and specificity of all three methods, including our new molecular workflow, by additionally diagnosing all cattle samples with the commercially-available coproELISA. Several studies have reported a decreased sensitivity of the coproELISA since commercialisation when diagnosing samples containing ≤10 EPG and using the manufacturers recommended positive threshold [15–16]. This is despite reporting detection limits of 0.6 ng/ml (cattle) and 0.3 ng/ml (sheep) of *Fasciola* spp. antigen, corresponding to a sensitivity of 100% for cattle harbouring 2 or more flukes, or sheep harbouring 1 fluke, during development [6]. In our study, samples <10 EPG were in agreement with previous reports, and in response we re-evaluated the positive threshold by employing several previously-described methods [13–16]. This re-evaluation increased the diagnostic sensitivity of the coproELISA, particularly in the recently infected herd (Herd 2). However, the application of arbitrary statistical methods to increase the sensitivity of the assay is problematic, as these methods are unlikely to be applicable across each new population being tested [13]. Hence, a new positive threshold must be calculated for each new batch number and species being diagnosed, resulting in additional costs and an unnecessary waste of time, particularly where large samples sizes are involved. Further, despite the added benefits of earlier diagnosis, the lack of ability to speciate still remains in regions such as Southeast Asia with both *Fasciola* spp. present. While still being limited by the pre-patent period, the new molecular workflow for the detection of *Fasciola* spp. in faecal samples maintains the larger throughput associated with the coproELISA at a similar cost, whilst providing the added benefit of species differentiation.

The new molecular workflow provides a simple step-wise process for the preparation of faecal samples enabling medium-high throughput for the diagnosis of Fasciolosis. However, the application of this approach is of limited use in locations where the diagnostic capacity may be restricted or if the necessary laboratory equipment is lacking. The ability to preserve samples in 70% EtOH and transport them to areas with increased diagnostic capacity is vital, particularly when conducting epidemiological studies on Fasciolosis in remote and rural areas such as Southeast Asia. The opportunity of sample preservation for transport has been demonstrated previously, particularly when working in areas lacking a continuous cold chain [23,31]. Our results were in agreement, demonstrating that regardless of the storage conditions we applied, there was no effect on sedimentation and DNA isolation.

The advancement of molecular tools for the differentiation of *Fasciola* spp. have greatly added to our understanding of their ecology, epidemiology and zoonotic potential [19]. However, no tool currently exists for the identification of the hybrids between *F*. *hepatica* and *F*. *gigantica* in the field. This is especially important in areas where *F*. *hepatica*, *F*. *gigantica* and their hybrids exist in sympatry, such as Southeast Asia, where the zoonotic potential of the hybrid forms are largely unknown [19, 32]. The inclusion of TaqMan probes in our new molecular diagnostic workflow enables the identification of either infection with a hybrid, or a mixed infection with both *F*. *hepatica* and *F*. *gigantica* within a single animal [10]. However, due to the triploid nature of the hybrids, the differentiation between these two scenarios would require the additional isolation of DNA from single eggs [33]. Our new molecular diagnostic workflow provides this capability, as demonstrated by the repeated successful isolation of DNA from single eggs of both *F*. *hepatica* and *F*. *gigantica*, adding an additional tool to our diagnostic arsenal.

In conclusion, we present a robust approach for the ante-mortem diagnosis of *Fasciola* spp. infection using faecal samples. The presented workflow is able to differentiate between *F*. *hepatica* and *F*. *gigantica* species, while also providing a flexible methodology capable of being adapted for use in existing diagnostic laboratory workflows. Although the method maintains the requirement for the completion of the pre-patent period, the additional benefits of fluke species differentiation and increased sample throughput provide clear benefits over the traditional sedimentation and FEC approach. The high diagnostic sensitivity and ability to store samples in 70% EtOH make this approach suitable for use in surveillance programs and epidemiological studies in areas where access to a complete cold chain is lacking or where laboratory capacity is limited.

## Supporting information

S1 Table Newcastle climate data. (XLSX)

S2 Table Percentage of eggs lost during sedimentation. (XLSX)

S3 Table Impact of faecal sample volume on DNA isolation. (XLSX)

S4 Table Diagnostic sensitivity and specificity. (XLSX)

S5 Table Impact of storage conditions on sedimentation outcomes and DNA isolation. (XLSX)

S1 Fig Removal of non-specific amplification with probes. (PDF)

S2 Fig No amplification of paramphistome DNA. (PDF)

S3 Fig No effect of storage time on positive/negative coproELISA outcomes. (PDF)

S4 Fig Egg and fluke corresponding values. (PDF)

S5 Fig Standard curve. (PDF)

S6 Fig STARD checklist. (PDF)

S7 Fig STARD flowchart. (PDF)
