# Supplementary material for: Scrambled eggs: A highly sensitive molecular diagnostic workflow for Fasciola species specific detection from faecal samples
Source: PLoS Negl Trop Dis. 2017 Sep 15;11(9):e0005931. doi: 10.1371/journal.pntd.0005931 (PMC5617325; doi:10.1371/journal.pntd.0005931)

Supporting Figure 1. Removal of non-specific background amplification with *Fasciola* species-specific TaqMan probes

A. Without probes (red – adult *F. hepatica* (positive control), green – faecal samples, blue – no template control, black – extraction control)

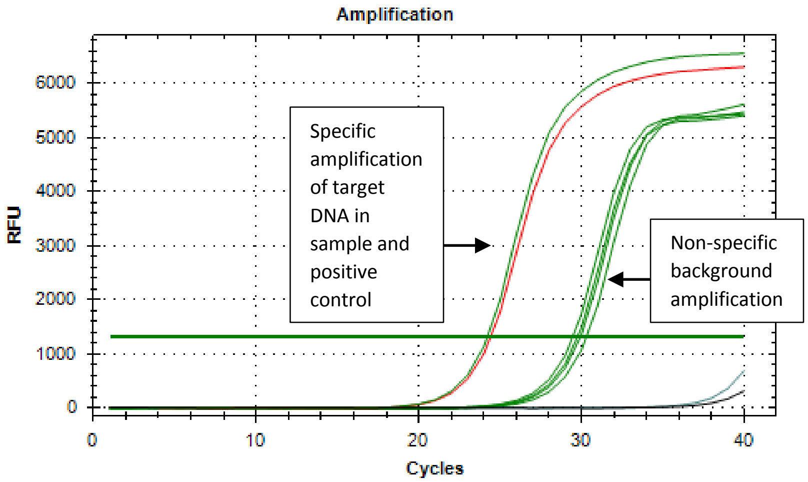

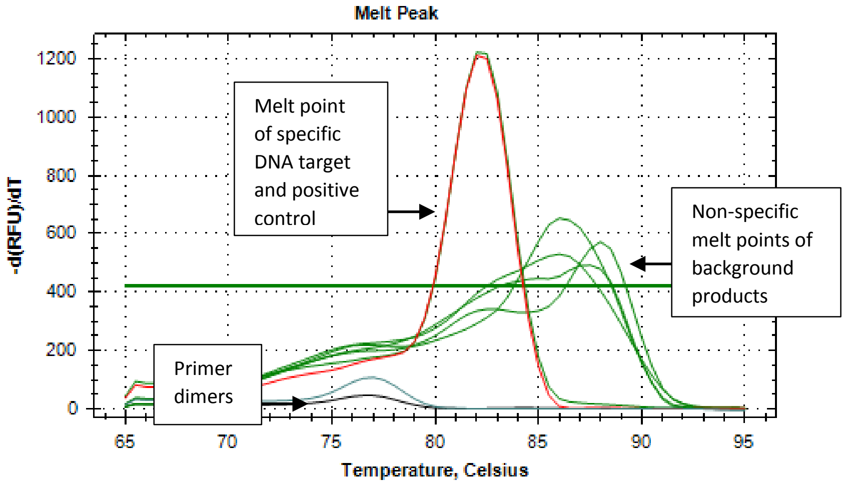

B. With probes (red – adult *F. hepatica* (positive control), green – faecal samples, blue – no template control, black – extraction control)

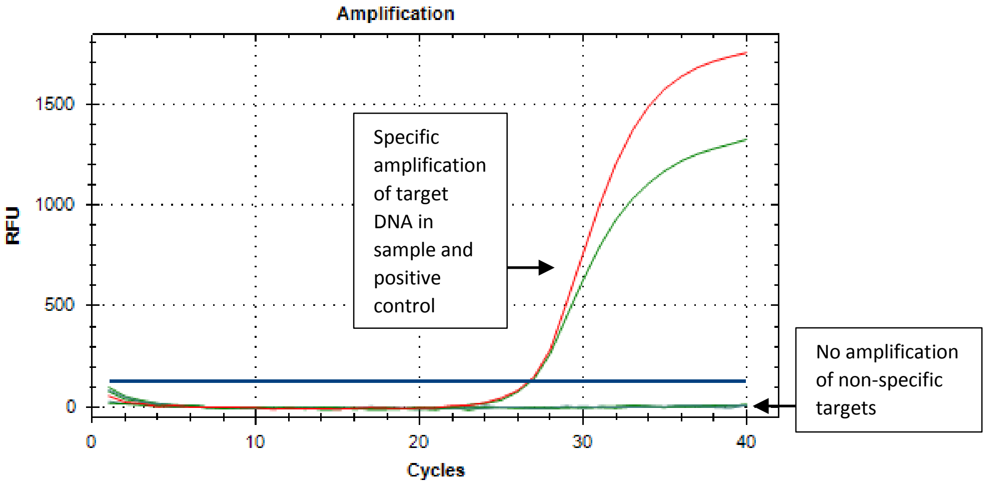

Supplement: S1 Fig — (PDF) [file pntd.0005931.s006.pdf]
