# Supplementary material for: Scrambled eggs: A highly sensitive molecular diagnostic workflow for Fasciola species specific detection from faecal samples
Source: PLoS Negl Trop Dis. 2017 Sep 15;11(9):e0005931. doi: 10.1371/journal.pntd.0005931 (PMC5617325; doi:10.1371/journal.pntd.0005931)
Supplement: S2 Fig — (PDF) [file pntd.0005931.s007.pdf]

Supporting Figure 2. No amplification of paramphistome DNA using *Fasciola* species-specific TaqMan probes

A. Amplification curve (red – positive control (adult *F. hepatica*), blue – paramphistome DNA isolated from 10-20 'grey-blue' eggs from an *F. hepatica* positive faecal sample, grey – no template control)

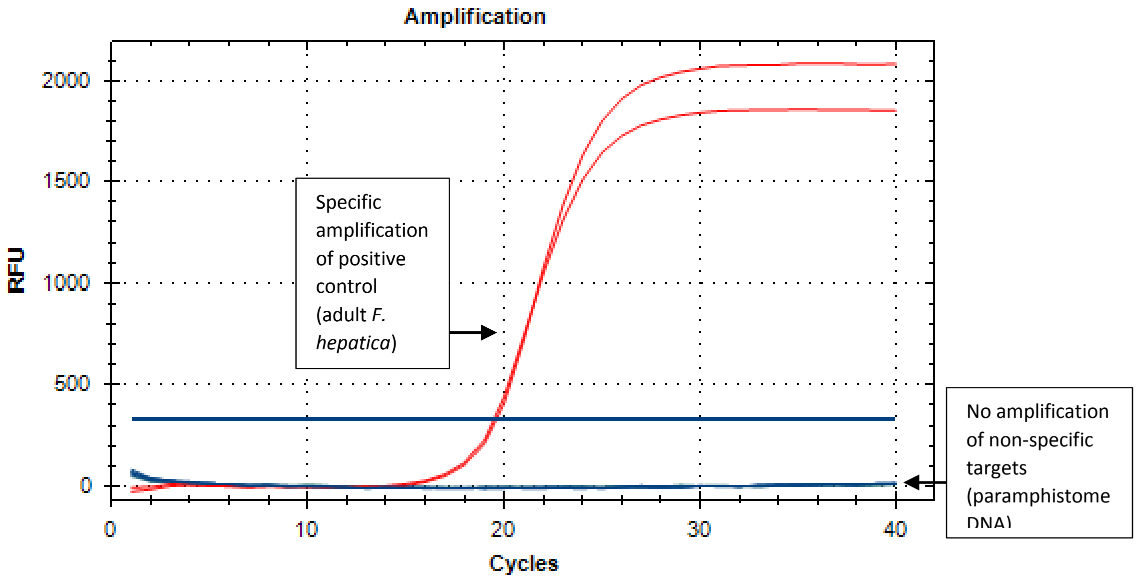

B. Quantification data (positive control – adult *F. hepatica*, negative control 3 – 20 paramphistome eggs isolated from an *F. hepatica* positive faecal sample (replicate 1), negative control 4 - 20 paramphistome eggs isolated from an *F. hepatica* positive faecal sample (replicate 2))

Well	Fluor	Target	Content	Sample	Cq
A01	FAM		Pos Ctrl-1	Fh 1:100	19.45
B01	FAM		Pos Ctrl-1	Fh 1:100	19.58
C01	FAM		NTC-2	ddH2O	N/A
D01	FAM		NTC-2	ddH2O	N/A
E01	FAM		Neg Ctrl-3	Param 1	N/A
F01	FAM		Neg Ctrl-3	Param 1	N/A
G01	FAM		Neg Ctrl-4	Param 2	N/A
H01	FAM		Neg Ctrl-4	Param 2	N/A
