# Supplementary material for: Scrambled eggs: A highly sensitive molecular diagnostic workflow for Fasciola species specific detection from faecal samples
Source: PLoS Negl Trop Dis. 2017 Sep 15;11(9):e0005931. doi: 10.1371/journal.pntd.0005931 (PMC5617325; doi:10.1371/journal.pntd.0005931)
Supplement: S3 Fig — (PDF) [file pntd.0005931.s008.pdf]

Supporting Figure 3. Storage condition has no impact on the positive/negative *F. hepatica* corpoELISA outcome

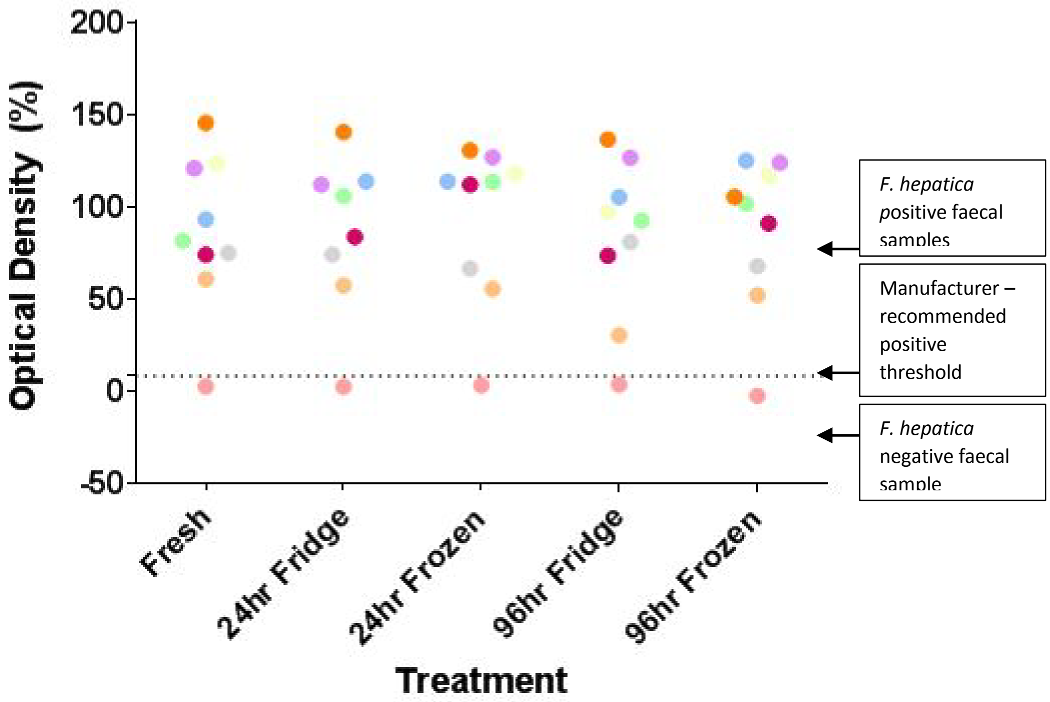

Colours indicate individual faecal samples subjected to five different storage conditions
