# Supplementary material for: Scrambled eggs: A highly sensitive molecular diagnostic workflow for Fasciola species specific detection from faecal samples
Source: PLoS Negl Trop Dis. 2017 Sep 15;11(9):e0005931. doi: 10.1371/journal.pntd.0005931 (PMC5617325; doi:10.1371/journal.pntd.0005931)
Supplement: S4 Fig — (PDF) [file pntd.0005931.s009.pdf]

Supporting Figure 4. Adult *F. hepatica* and 2000 eggs corresponding values

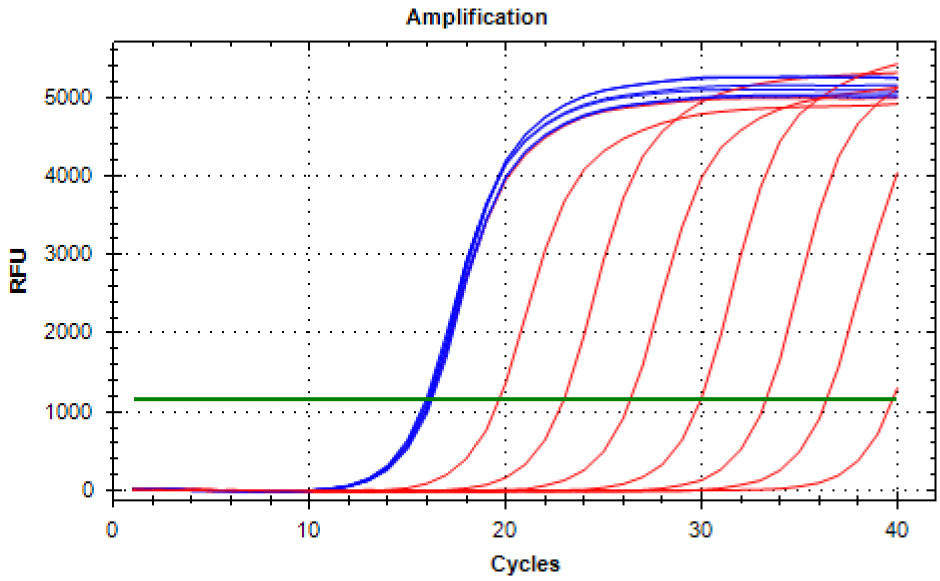

Blue – 2000 clean *F. hepatica* eggs subjected to 40 seconds of disruption at 6.0 m/s, red – adult *F. hepatica* 10-fold serial dilution
