# Supplementary material for: Scrambled eggs: A highly sensitive molecular diagnostic workflow for Fasciola species specific detection from faecal samples
Source: PLoS Negl Trop Dis. 2017 Sep 15;11(9):e0005931. doi: 10.1371/journal.pntd.0005931 (PMC5617325; doi:10.1371/journal.pntd.0005931)
Supplement: S5 Fig — (PDF) [file pntd.0005931.s010.pdf]

Supporting Figure 5. 10-fold dilution of *F. hepatica* DNA from adult fluke samples

A. Amplification curve

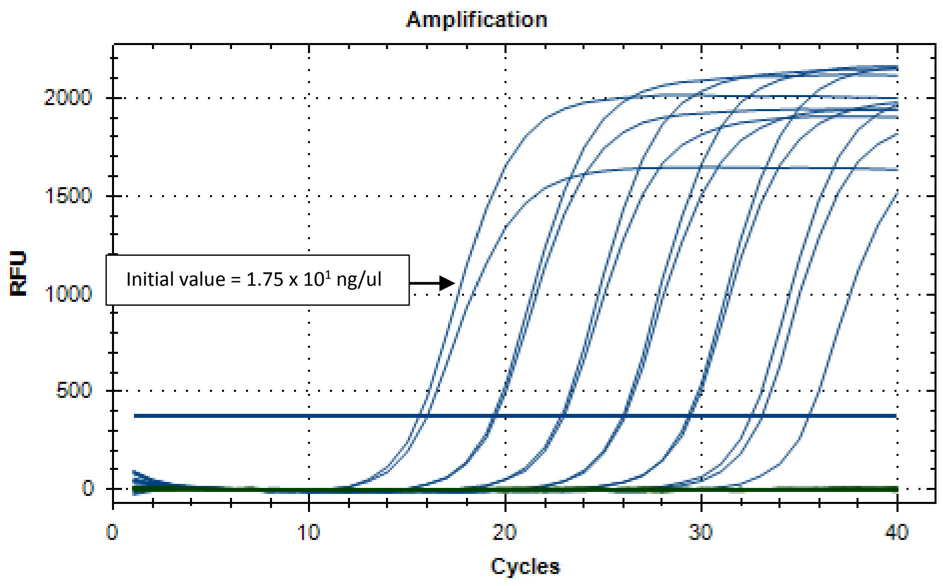

B. Standard curve

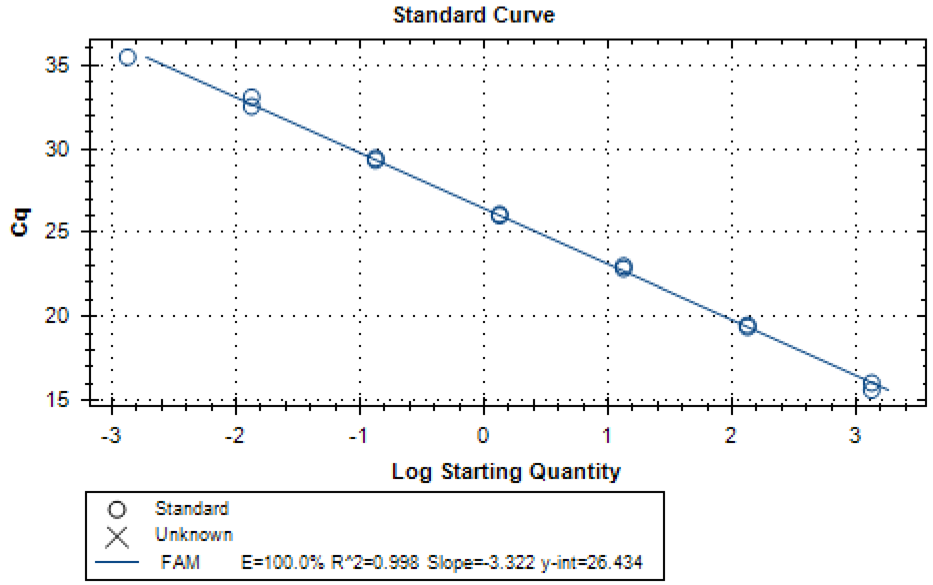

C. Quantification data

Well	Fluor	Target	Content	Sample	Cq	Cq Mean	Cq Std. Dev	Starting Quantity (SQ)	Log Starting Quantity	SQ Mean
A12	FAM		NTC	ddH2O	N/A	0.00	0.000	N/A	N/A	0.00E+00
B01	FAM		Std	F1:10	15.60	15.60	0.000	1.333E+03	3.125	1.33E+03
B12	FAM		Std	F1:10	16.03	16.03	0.000	1.333E+03	3.125	1.33E+03
C01	FAM		Std	F1:100	19.37	19.37	0.000	1.330E+02	2.124	1.33E+02
C12	FAM		Std	F1:100	19.49	19.49	0.000	1.330E+02	2.124	1.33E+02
D01	FAM		Std	F1:1000	22.83	22.83	0.000	1.330E+01	1.124	1.33E+01
D12	FAM		Std	F1:1000	23.00	23.00	0.000	1.330E+01	1.124	1.33E+01
E01	FAM		Std	F1:10^4	26.01	26.01	0.000	1.330E+00	0.124	1.33E+00
E12	FAM		Std	F1:10^4	26.09	26.09	0.000	1.330E+00	0.124	1.33E+00
F01	FAM		Std	F1:10^5	29.33	29.33	0.000	1.330E-01	-0.876	1.33E-01
F12	FAM		Std	F1:10^5	29.45	29.45	0.000	1.330E-01	-0.876	1.33E-01
G01	FAM		Std	F1:10^6	32.54	32.54	0.000	1.330E-02	-1.876	1.33E-02
G12	FAM		Std	F1:10^6	33.10	33.10	0.000	1.330E-02	-1.876	1.33E-02
H12	FAM		Std	F1:10^7	35.49	35.49	0.000	1.330E-03	-2.876	1.33E-03
