# Supplementary material for: Scrambled eggs: A highly sensitive molecular diagnostic workflow for Fasciola species specific detection from faecal samples
Source: PLoS Negl Trop Dis. 2017 Sep 15;11(9):e0005931. doi: 10.1371/journal.pntd.0005931 (PMC5617325; doi:10.1371/journal.pntd.0005931)
Supplement: S6 Fig — (PDF) [file pntd.0005931.s011.pdf]

Section & Topic	No	Item	Reported on page #
**TITLE OR ABSTRACT**			
	**1**	Identification as a study of diagnostic accuracy using at least one measure of accuracy (such as sensitivity, specificity, predictive values, or AUC)	1, 3
**ABSTRACT**			
	**2**	Structured summary of study design, methods, results, and conclusions (for specific guidance, see STARD for Abstracts)	2, 3
**INTRODUCTION**			
	**3**	Scientific and clinical background, including the intended use and clinical role of the index test	4, 5, 6
	**4**	Study objectives and hypotheses	5, 6
**METHODS**			
Study design	**5**	Whether data collection was planned before the index test and reference standard were performed (prospective study) or after (retrospective study)	7
Participants	**6**	Eligibility criteria	7
	**7**	On what basis potentially eligible participants were identified (such as symptoms, results from previous tests, inclusion in registry)	7
	**8**	Where and when potentially eligible participants were identified (setting, location and dates)	7
	**9**	Whether participants formed a consecutive, random or convenience series	7
Test methods	**10a**	Index test, in sufficient detail to allow replication	14
	**10b**	Reference standard, in sufficient detail to allow replication	8
	**11**	Rationale for choosing the reference standard (if alternatives exist)	17
	**12a**	Definition of and rationale for test positivity cut-offs or result categories of the index test, distinguishing pre-specified from exploratory	15
	**12b**	Definition of and rationale for test positivity cut-offs or result categories of the reference standard, distinguishing pre-specified from exploratory	8, 9
	**13a**	Whether clinical information and reference standard results were available to the performers/readers of the index test	n/a
	**13b**	Whether clinical information and index test results were available to the assessors of the reference standard	9
Analysis	**14**	Methods for estimating or comparing measures of diagnostic accuracy	17
	**15**	How indeterminate index test or reference standard results were handled	n/a
	**16**	How missing data on the index test and reference standard were handled	n/a
	**17**	Any analyses of variability in diagnostic accuracy, distinguishing pre-specified from exploratory	19, 21
	**18**	Intended sample size and how it was determined	7
**RESULTS**			
Participants	**19**	Flow of participants, using a diagram	14
	**20**	Baseline demographic and clinical characteristics of participants	7
	**21a**	Distribution of severity of disease in those with the target condition	n/a
	**21b**	Distribution of alternative diagnoses in those without the target condition	n/a
	**22**	Time interval and any clinical interventions between index test and reference standard	7
Test results	**23**	Cross tabulation of the index test results (or their distribution) by the results of the reference standard	20
	**24**	Estimates of diagnostic accuracy and their precision (such as 95% confidence intervals)	20
	**25**	Any adverse events from performing the index test or the reference standard	n/a
**DISCUSSION**			
	**26**	Study limitations, including sources of potential bias, statistical uncertainty, and generalisability	29
	**27**	Implications for practice, including the intended use and clinical role of the index test	29
**OTHER INFORMATION**			
	**28**	Registration number and name of registry	n/a
	**29**	Where the full study protocol can be accessed	17
	**30**	Sources of funding and other support; role of funders	Submitted online

cc BY SA
