# Supplementary figures and images for: Scrambled eggs: A highly sensitive molecular diagnostic workflow for Fasciola species specific detection from faecal samples
Source: PLoS Negl Trop Dis. 2017 Sep 15;11(9):e0005931. doi: 10.1371/journal.pntd.0005931 (PMC5617325; doi:10.1371/journal.pntd.0005931)

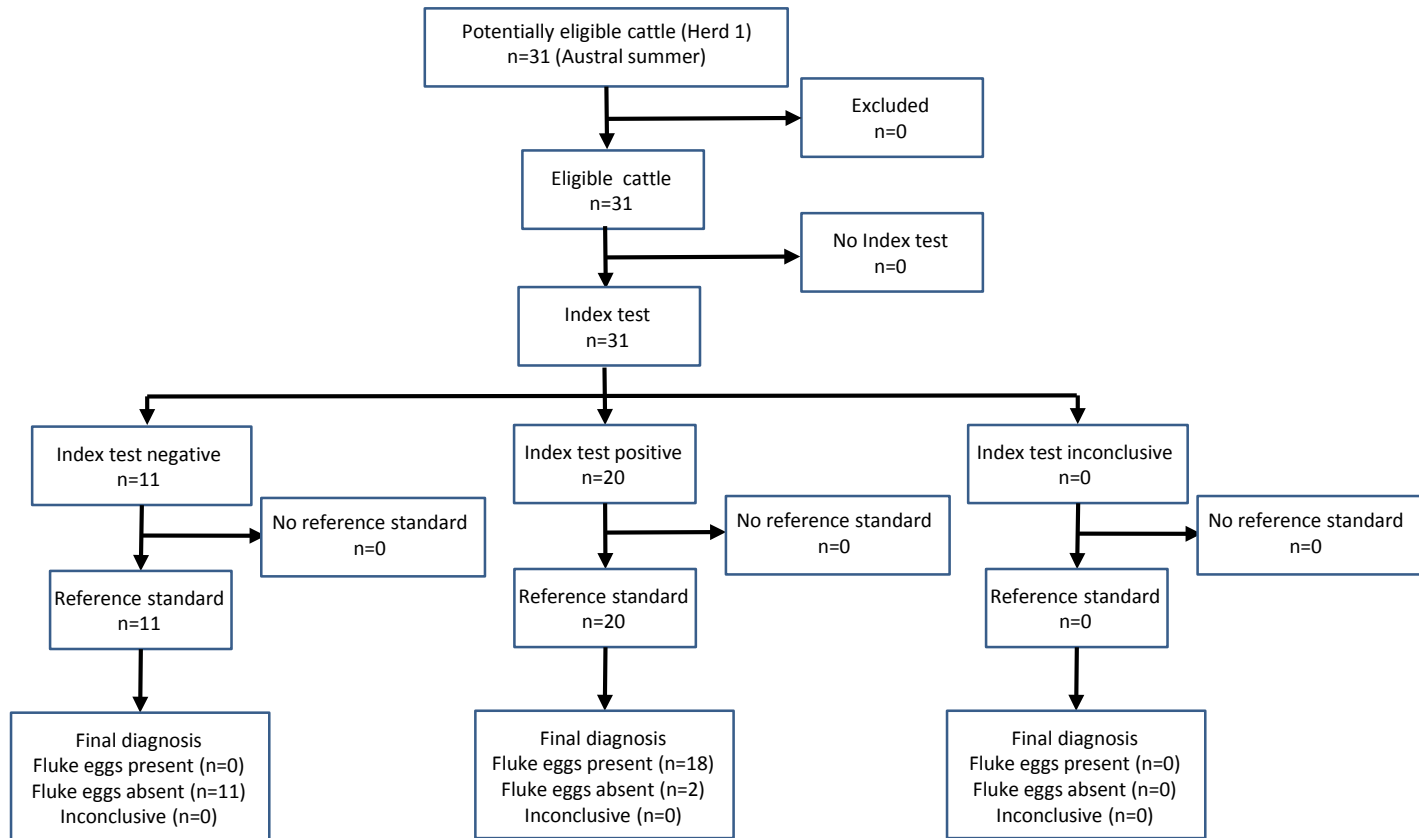

Supplement: S7 Fig — (PDF) [file pntd.0005931.s012.pdf]
